# Hybrid procedures in treatment of multilevel lesions of the brachiocephalic arteries

**DOI:** 10.1186/1753-6561-7-S1-P5

**Published:** 2013-01-30

**Authors:** Goar Arutyunyan, Elena Annenkova, GV Sinyavin

**Affiliations:** 1 IM Sechenov First Moscow Medical State University, Moscow, Russia

## Background

Multilevel significant atherosclerotic disease involving the carotid bifurcation and the proximal ipsilateral common carotid or brachiocephalic arteries presents an uncommon and difficult management problem. Axial (aortocarotid bypass) or extra-anatomic procedures (subclavian–carotid bypass, carotid–carotid bypass, carotid–subclavian transposition) and variations in the performance of thromboendarterectomies are all possibilities in treatment of tandem brachiocephalic arteries stenosis. Although extra-anatomic procedures have excellent patency (90% at 5 years) and durability and avoid the morbidity of median sternotomy, they are usually performed in patients with significant cardiovascular comorbidities who may be detrimentally affected because of the use of general anesthesia and the possible need of prosthetic devices and multiple incisions. The desire to avoid the need for intrathoracic and extra-anatomic reconstructions has fostered the use of transluminal angioplasty and primary stenting of the supra-aortic vessels. Combined or hybrid carotid procedures can offer an easier solution to a sometimes difficult technical problem.

## Methods

The aim of this study was to review the existing literature on such hybrid procedures. An electronic search of the pertinent literature was undertaken.

## Results

Grego et al. treated 16 patients with CEA and retrograde angioplasty and stenting for tandem lesions. The procedure was successful in 14, and there was no neurologic morbidity or mortality at 30 days. At 1-year follow-up, all treated vessels were patent and patients were free of focal cerebrovascular symptoms 1 In 2011 Karpenko AA et al. shared their experience with hybrid surgical interventions for multilevel lesions of the brachiocephalic arteries (stenting and an open operation) in a total of 11 patients presenting with cerebrovascular insufficiency. No intraoperative complications were encountered 2. In 2011 a meta-analysis of all studies reporting on simultaneous carotid endarterectomy and retrograde angioplasty for the treatment of tandem internal carotid and proximal common carotid or innominate artery lesions was performed. 13 studies, including 133 patients were identified. Reported technical success of the procedure was 97%. This meta-analysis reports the largest collection of patients having undergone hybrid treatment of tandem disease of the arch vessels and carotid bifurcation. Results from this study show that the combined stroke and death rate with this approach is equal to or better than that for isolated endarterectomy 3.

## Conclusions

We have demonstrated that CEA combined with proximal stenting seems a simple and convenient alternative to conventional surgery for symptomatic multilevel disease of the extra-cranial vessels. We suggest this procedures will become the treatment of choice for this subset of patients.

## References

[B1] Akers DL Markowitz IA Kerstein MD The value of aortic arch study in the evaluation of cerebrovascular insufficiency Am J Surg 1987 154 230 2 10.1016/0002-9610(87)90187-5 3631398

[B2] Levien LJ Benn CA Veller MG Fritz VU Retrograde balloon angioplasty of brachiocephalic or common carotid artery stenoses at the time of carotid endarterectomy Eur J Vasc Endovasc Surg 1998 15 521 7 10.1016/S1078-5884(98)80113-5 9659888

[B3] Grego F Frigatti P Lepidi S Bonvini S Amista P Deriu GP Synchronous carotid endarterectomy and retrograde endovascular treatment of brachiocephalic or common carotid artery stenosis Eur J Vasc Endovasc Surg 2003 26 392 5 10.1016/S1078-5884(03)00323-X 14512001

